# Foreign Summary

**Published:** 1839-12-21

**Authors:** 


### FOREIGN SUMMARY.

### Velpeau's Clinical Lectures on Ophthalmia.

### No. XII.

#### SEQUELÆ AND COMPLICATIONS OF KERATITIS.

*Description of different kinds of opacity of the cornea.—Treatment.—Dieffenbach's operation.—Iritis.—Historical detail regarding the disease, first described by the German and English surgeons.—Acute iritis.—Symptoms.—The various forms of iritis.—Causes of acute iritis.*

Ulceration of the cornea is always followed by a certain degree of opacity of that membrane. This opacity may be compared to the cicatrix which follows the ulceration of other membranes, and as it generally more or less impairs the visual functions, it has attracted much attention. Authors have described many kinds of opacity, but they may be all comprised in three species: the *nebula*, in which the opacity occupies only the superficial lamellæ of the cornea; *albugo*, in which the middle layers are also affected; and lastly, *leucoma*, in which the entire thickness of the cornea has become opaque. I may also mention another species of opacity which has been but little noticed by authors, probably because it offers no impediment to sight, the *senile zone* or *ring*.

The *nebula* consists in a slight opacity of the superficial lamellæ of the cornea. The word itself indicates the nature of the opacity; it may be compared to a slight mist or cloud, obscuring, but not destroying the transparency of the membrane. This lesion is often confounded with the nephelion, or superficial ulcer of the cornea, the achlys of Galen, which, as you will remember, rests on a slight nebula. We may thus account for Scarpa's asserting that a nebula is always accompanied by slight vascularization of the conjunctiva, my own experience enabling me to say, that when the nebula is unaccompanied by ulceration of the cornea, the vascular appearance noted by Scarpa does not exist.

An albugo is an opacity of sufficient density to intercept the passage of light through that part of the cornea on which it is situated. The principal distinction between albugo and leucoma is, that albugo does not occupy the entire thickness of the membrane. It may be situated in any part of the cornea, is of a milky yellowish colour, and is met with as a sequela of intense chronic keratitis, of abscess, or of ulceration of the cornea.

*Leucoma*, generally the result of a cicatrix, of lengthened suppuration, or of intense inflammation, is not merely characterized by opacity of the entire membrane, but also by other modifications which the texture of that membrane undergoes. It becomes hard, callous, thickened, and its vitality is diminished.

The *senile zone* is a circular, opaque ring, natural in old people, occupying that part of the cornea which is immediately adjoining to the sclerotica. It cannot be attributed, properly speaking, to disease of the cornea, the structure of that membrane not being altered, nor its thickness increased; and as the functions of vision are not impaired, little or no attention is, in most instances, paid to its presence. This form of opacity, extremely common in old age, is occasionally met with in all other periods of life. It generally commences on the superior portion of the cornea, then invades the lower hemisphere, and it is only as the patient advances in years that the two arcs join laterally. With old people, the opaque ring is always separated from the sclerotica by a small interval of transparent tissue; but this is not constantly the case in younger persons.

*Treatment.*—The treatment of the various species of opacity of the cornea has been much studied by surgeons; nor need we be surprised, when we consider that the slightest nebula situated opposite the cornea, must, more or less, disorder the visual functions. The non-medical public entertain singular notions respecting the nature and the treatment of these lesions; nothing, indeed, is more common than to hear patients affected with opacity of the cornea complain of having a membrane over the eye, which they wish to be taken off. However ridiculous the idea may appear, we shall see presently that such an operation has not only been proposed, but even put into execution by some practitioners. You must remember that the public in general know nothing whatever about medicine, and that all the absurd ideas respecting the treatment of disease which are current in the world, have originated with medical men. Even those remedies which we call "old women's remedies" have been at one time employed by the profession; thus you will find that cow-dung, which they advise in inflammatory humours, is greatly extolled by Avicenna.

In most instances, opacities of the cornea are indelible; there is nothing to be done. Indeed the attempts which are made to remove an albugo, or a leucoma, often increase the gravity of the lesion. When, however, the opacity is merely a nebula, and one or two of the superficial lamellæ of the cornea only are affected, there is some chance of being able either entirely to remove it, or at least to increase the transparency of the membrane. The white matter, combined with the tissue of the cornea, instead of the transparent fluid which it generally contains, and the remedies which are employed being applied to the surface of the affected membrane, may penetrate and modify the nature of the effused matter. Among the therapeutic agents which are the most frequently resorted to with this view, are the various collyria employed in the treatment of ulcers of the cornea; as also the sulphate of zinc, calomel in powder, the solid nitrate of silver, Sydenham's laudanum, &c.

Some of these remedies, such as laudanum and the lunar caustic, enjoy greater reputation than others. They certainly may sometimes be used with success to dispel slight nebulæ, when there is no inflammation; but if they are employed whilst the inflammation of the cornea still exists, they nearly always occasion a renewal of the ophthalmia. The nitrate of silver, used as a caustic, was much praised, a few years back, by M. Lallemand, who advises the opacity to be slightly cauterized five or six times in the course of the month. I think, however, he must have mistaken for a nebula, the superficial ulcer of the cornea or nephelion, against which cauterization generally proves successful. I have several times tried the nitrate of silver in the treatment of nebulæ, and constantly seen the malady exasperated.

In the last century, a surgeon, named Gouan, pretended he could cure nearly every species of opacity of the cornea with a liquid, the composition of which he kept secret. The secret was bought up, and this marvellous remedy turned out to be merely oil of walnuts. To support his assertions respecting its efficacy, he appealed to the authority of Linnæus, as also to the customs of the inhabitants of the Pyrenees, who had employed it against opacities of the cornea from time immemorial. The remedy had been long forgotten, when, a short time since, it was again brought forward by M. Carron-du-Villiards, who makes use, however, of cod-oil, and not of walnut-oil. I will not take upon me to decide which is to be preferred; yet I must say it appears to me, that, if one is an efficacious remedy, the other cannot be much inferior. With regard to the therapeutic properties of the oil, I do not believe that it can cure any other form of opacity than the nebula.

Some surgeons have advised that the opacity, when it does not occupy the entire thickness of the membrane, be excised, or gradually worn away by scraping; but there are no facts before the profession to prove that such an operation has ever been successfully performed. Whether the opacity be worn away or excised, there must evidently remain an epicauma ulcer on the surface of the cornea, which, on cicatrizing, would itself give rise to opacity. The only case in which scraping or excision could be excused, is that in which a plastic ulcer leaves behind it, on the cornea, some coagulated lymph; and even then, I would not myself resort to such a measure.

According to Mr. Jacob, of Dublin, the use of the nitrate of silver in the treatment of ulcers of the cornea is often followed by a blue coloration of the cicatrix; but the cases he brings forward do not, in my opinion, satisfactorily prove the fact. I have myself employed the nitrate of silver as a solution, as an ointment, or alone, in many hundred cases, without ever observing such a coloration. I know not, therefore, what to say, and can only explain the fact by supposing that the nitrate of silver must act differently in England to what it does in France.

The excision of the plexus of varicose vessels which often corresponds to the affected part of the cornea, has been much lauded by some surgeons, and especially by Scarpa, whilst others, on the contrary, look upon the operation as nearly useless. This diversity of opinion may, however, easily be explained. When the opacity is kept up by the deep-seated or sclerotic vessels, excision is useless; but when it is the conjunctival vessels only that are morbidly developed, their section may produce, as Scarpa says, extraordinary results. This we have already seen to be the case when excision is performed in chronic keratitis, and in ulcers of the cornea.

As a last resource, when an albugo or a leucoma exists which entirely intercepts the passage of light into the eye, a singular remedy has been proposed—the excision of the affected portion of the cornea, and the subsequent union of the lips of the wound by suture, or the substitution of the cornea of some animal. M. Dieffenbach, a surgeon in every respect worthy of confidence, told me that he had successfully performed the operation on a young girl seven years old. Having fixed the cornea with a small tenaculum, he excised at once the affected portion. M. Dieffenbach also said that he thought an opaque cornea might be replaced by that of an animal. This operation was formerly proposed by Pellier, who even says that he had successfully performed it; but I do not place much reliance on his statement, for he was a specialist, and those gentlemen have generally something more in view then the interest of science. The operation appears to me, I must confess, perfectly absurd; indeed, I am at a loss to conceive how it could originate in a properly organised mind.

*Iritis.*

It is believed by many that iritis is a disease known only to modern writers; but if we peruse the treatises on ophthalmology which are anterior to the present century, we shall find that the existence of inflammation of the iris has always been recognised, although it is only since the commencement of the nineteenth century that it has been considered apart from the other inflammatory affections of the eye. Some authors had, it is true, done more than merely mention the existence of iritis, but as they confounded it with various diseases of the eye, the descriptions they give are vague and unsatisfactory. The Germans and the English were the first to separate iritis from all other ocular affections, and to make a distinct disease; but in doing so they have gone rather too far, their views being in my opinion too exclusive. Iritis exists, most certainly, as a distinct affection, and ought to be described as such, but, owing to the anatomical situation of the iris, it is hardly possible that it should be inflamed without the inflammation extending more or less to the adjoining tissues. It is, therefore, evidently necessary, in describing the malady, to take into consideration the various complications that may occur. At a future period we may be able to isolate completely iritis from all other inflammatory affections, but in the present state of science it is quite impossible to do so. Those, indeed, who have attempted it have invariably confounded, under the name of iritis, several inflammatory diseases which cannot in reality be referred to the iris.

Although it be to the German and to the English surgeons that we owe the first accurate description of iritis, you must not suppose that the affection was previously unknown to French writers. Maître Jean speaks of it in very plain terms; Deshais-Gendron describes it in treating of the iris; Janin published a case of iritis in his work on ophthalmology, and several other authors have spoken more or less briefly on the subject. Beer, is, however, the first who carefully described iritis. Two years later Schmidt published an interesting memoir on the disease in question, and nearly about the same time Ware and Saunders, in England, and at a later period, Mr. Travers made known the results of their researches. During the first twenty years of the present century France did but little towards the advancement of this branch of ophthalmology, but in 1820 M. Guillé (Dict. des Sciences Médic.) and M. Müller (Bibli. Ophthal.) entered the arena, and in 1823, M. Gillet de Grammont gave in his thesis as good a description of the disease as can be met with in the works of any foreign author. Since then many surgeons have written on the subject.

From this slight historical sketch, it appears that iritis has long been known to French writers, who, however, confounded it with various inflammatory affections of the eye, but that it is to foreigners that is due the credit of having first carefully studied the malady, and separated it from other diseases. It yet remains to be proved whether, by passing from one extreme to the other, the latter have not to a certain extent impeded the progress of science.

On looking over the different statistical accounts of diseases of the eye that have been published, we are at first surprised to find that iritis is by no means as common with some practitioners as with others. The difference of opinion which still exists respecting this malady will, however, at once account for the circumstance. It is, indeed, extremely difficult to limit iritis, as many of the symptoms which are referred to it by some writers are attributed by others to inflammation of the chlorid membrane, of the retina of the vitreous humour, or of the capsule of the crystalline lens.

I do not flatter myself that I shall be able completely to solve this pathological question, but as I have seen a great deal of the disease, and have attentively studied its characteristic symptoms, as also those of the inflammatory affections by which it is generally accompanied, the description which I am about to give may throw some additional light on the subject, and will, I think, give you more correct knowledge of the malady than can be gathered from our classical works on ophthalmology. There is, however, still much to be done before we can be said to have attained a perfect knowledge of this department of ocular pathology, to which I cannot too strongly recommend practitioners to direct their attention.

Iritis may, like all other inflammatory affections, be either acute or chronic. We will first study the acute form of the disease.

*Acute Iritis.*

Acute inflammation of the iris may commence either by the anterior or serous, or by the posterior or uveal surface of the organ, or by its parenchyma. I shall, however, lay but little stress on the symptoms which distinguish these shades of inflammation, as in the present state of science the distinction can be but of very slight importance in a practical point of view. Indeed, in a membrane so thin as the iris, inflammation cannot long remain confined to one region; it must at an early period invade the entire membrane.

Acute iritis may be considered to present three stages. Were there but few symptoms present, such a division would be useless; but as the symptoms are on the contrary extremely numerous, it will prove an useful adjuvant to the memory.

*Symptoms.*—The symptoms of iritis are generally divided into two classes—the anatomical and the physiological. The anatomical symptoms are those that can be appreciated by the eye of the surgeon; the physiological symptoms are those that depend on functional disorder of the organ, most of which can only be appreciated by the patient himself.

The *anatomical* or *objective* symptoms are numerous, and may also be divided into two classes; those which are to be referred to the inflamed organ, and those which are furnished by the adjoining tissues; such as the sclerotica, the cornea, the conjunctiva, the anterior chamber, &c. The symptoms which are given by the iris itself are more difficult to appreciate than those which we observe in keratitis; they are, indeed, in many instances, so fugacious, that until you have often seen the disease you will find it difficult to recognise their presence. They depend on the diameter of the pupil, on its form, its mobility, its colour, its thickness, and on the appearance which its surface presents. In the first stage of the disease, which may be said to extend over the two or three first days, the pupil contracts slightly, and loses in some measure its mobility, without, however, there being any visible alteration in its form. When the inflammation occupies one eye only, you will at once perceive the change that has taken place by comparing it with the other; but when both are affected it becomes extremely difficult to discover whether the pupil is or is not contracted; and were there no other symptom, the diagnosis would be very uncertain. As, however, the mobility of the pupil is always impaired when the iris is inflamed, it is possible to ascertain the real state of that organ by exposing rapidly to the light the eye, previously obscured for a few seconds. If the iris is healthy it immediately contracts; if, on the contrary, it is the seat of inflammation, contraction does not take place, or is very imperfect. As the inflammation increases, the contraction of the pupil appears more decided; it becomes at the same time perfectly immoveable, and the various preparations of belladonna lose nearly entirely the influence they exercise over its motion in the healthy state. The colour of the iris also undergoes modifications which ought to be attentively studied, although they are not of the extreme importance which they are supposed to be by the German ophthalmologists. The iris presents three zones or rings, one of which is situated near the pupillary or inner circumference of the organ, another near the ciliary or outer circumference, and the third is intermediate. The colour of the pupillary zone becomes modified before that of the external and intermediate zones, the nature of the change which takes place varying with the natural colour of the iris. Thus, it assumes a greenish tint when the iris is naturally blue; becomes, on the contrary, brown when the iris is gray, and brick-red when the iris is brown. These various hues also soon become visible on the ciliary zone. Between the two may be seen a network formed by numerous small vessels, which converge as they pass from the outer to the inner zone, and are crossed transversely by other vascular filaments. When the inflammation is acute, these vessels become more and more injected, and form a red zone between the two, which I have just described.

The texture of the iris is also modified, that organ becoming turgid, and thicker than in the natural state. Its anterior surface, which, when healthy, is smooth and polished, seems as if it had been macerating in some fluid, the small furrows which it presents disappearing, or otherwise it assumes a velvety appearance, and presents numerous specks of different colours.

The iris itself is not the only organ that is modified when it is the seat of inflammation; the adjoining tissues always undergo more or less alteration. The transparency of the cornea is slightly impaired. The sclerotica presents the vascular radiated zone which I have already described as a symptom of keratitis; but it is much less manifest than in than affection, unless, however, the iritis be accompanied by inflammation of the cornea. This radiated zone does not advance on the sclerotica as far as the cornea, from which it is separated by the arthritic circle. The conjunctiva is in general but very slightly injected.

The physiological or subjective symptoms are also of great importance in forming our diagnosis of the disease. From the first the patient feels pain, more or less severe, in the orbit, as also in the forehead and temples. If the inflammation becomes intense, he often complains of throbbing in the eye, and of an extremely painful sensation of distention of that organ. Fear of light, and shedding of tears, are also observed at an early period of the disease, but these symptoms are seldom as intense as in some other inflammatory affections of the eye, as, for instance, in ulcerated keratitis. M. Suhel does not allow that photophobia and epiphora are symptomatic of iritis, and says that when they are present it is because the iritis is accompanied by retinitis. But his views on this subject are certainly erroneous; it is extremely rare to meet with a case of iritis that does not present photophobia and epiphora to a certain extent: as might be anticipated, from the anatomical symptoms of the malady, vision is always more or less disordered.

The general symptoms in iritis are very variable: sometimes there is no reaction whatever; sometimes, on the contrary, there is fever, thirst, loss of appetite, &c.

The second stage of the disease is characterized by the exacerbation of all the symptoms which I have enumerated; the diagnosis, therefore, becomes much easier. The contraction of the pupil gradually increases; and, losing its circular form, it assumes an angular shape. This change in the form of the iris constitutes one of the surest and most convincing proofs of the existence of iritis, and when it is observed it is next to impossible not to recognise the nature of the disease. We cannot, therefore, be surprised that German ophthalmologists should have made it one of the principal characters by which the various specific forms of iritis which they describe may be distinguished from one another. When the form of the pupil is thus altered, it may represent a lozenge, an oval, an ellipsis; indeed, it may assume nearly every possible variety of shape, the modifications which it thus undergoes depending, according to their ideas, on the cause which has produced the iritis. Were this opinion correct, we should be able, by the examination of the eye alone, to say that the iritis has been produced by such or such a cause, and that it belongs to such or such a form of inflammation. But this is not in reality the case; the changes which occur in the form of the iris have no connection whatever with the cause that has given rise to the iritis; they are merely to be attributed to the tumefaction of one region of the iris being greater than that of another region, or to adhesions having been formed, which impede its movements. We have now in our wards four or five patients labouring under iritis who present this symptom, yet you will not find one out of the five with whom the iritis can be possibly ascribed to the cause which ought to have produced it, were the views of the German school on this subject correct. Indeed, the pupil of one of them has presented successively, in the course of a few days, all the principal modifications of shape which characterize the four chief forms of specific iritis—that is, the syphilitic, the scrofulous, the rheumatic, and the arthritic forms of that disease; and as we have continually facts of this kind under our eyes, it is really impossible to admit that the form which the pupil assumes, when inflamed, depends on a specific cause.

The humours of the eye losing their transparency, the colour of the pupil changes. Flakes or filaments of coagulable lymph often make their appearance, extending from one part of the pupillary circumference of the iris to another, and interlacing, so as to form all kinds of figures. Sometimes these flakes are only partly attached to the inner margin of the iris, or even float quite free in the pupil. It is nearly always very difficult to bring about the absorption of the lymph which has thus been effused, and as by its presence in the pupil it intercepts the entrance of light into the eye, the functions of vision are materially impaired. There can, in my opinion, be no doubt that these changes in the transparency of the pupil are due to the effusion of plastic lymph, which takes place as a consequence of the inflammation of the iris. This membrane, situated as it is in the centre of the anterior chamber, may be compared to a fire placed in the middle of a room, the caloric emanating from which must necessarily modify the bodies that surround it.

In some instances, the naturally smooth surface of the iris presents numerous folds or furrows, as also well-defined specks and patches, which are to be attributed to ecchymosis, or to the effusion of purulent matter into the tissue of the organ. The patches are slightly raised above the level of the surrounding parts of the iris, and are encircled by a yellow areola. The specks are either of a brown or of a yellow colour, and protrude more or less into the anterior or the posterior chamber. When these symptoms are observed the iris seldom retains its usual situation ; it is generally pushed either forwards towards the cornea, or backwards towards the crystalline lens. This phenomenon has been described under the name of *synechia*, anterior and posterior, and is caused by an increased secretion of the humours of the eye. Thus, when the uveal surface of the iris is first inflamed, the aqueous humour in the posterior chamber is increased, and the iris being pushed forwards, anterior synechia is produced; this also occurs when the volume of the vitreous humour is augmented. When it is the anterior surface of the iris that is first inflamed, the aqueous humour in the interior chamber is increased, and the iris being pushed backwards, it is posterior synechia which is produced. Synechia may also be caused by the adhesions which the iris contracts with other parts of the eye. It will be anterior when it adheres to the cornea, posterior when it adheres to the crystalline lens.

When the iritis is intense, in this stage of the disease the inflammation often extends to the conjunctiva. The transparency of the cornea becomes more and more impaired, and the radiated sclerotic zone appears wider and of a deeper red. In some instances this zone advances as far as the cornea, the vessels by which it is formed losing themselves in the superficial lamellæ of that organ; in others, it stops at the union of the cornea with the sclerotica, and in others again it remains separated from the cornea by a gray or blue ring, as in the first stage.

At this period the pain felt in and about the eye may become very severe. It appears to extend more especially in the direction of the branches of the facial nerve, and of the fifth pair. The photophobia and the epiphora generally increase; sometimes, on the contrary, they become less intense. The exacerbation of the latter symptoms is occasionally accompanied by fever, nausea, and even delirium, but this is not often the case. It is worthy of remark, that there is seldom much general reaction in iritis, even when the inflammation is violent. Iritis differs in this respect from conjunctivitis and keratitis.

In the third stage of the malady we meet with all the symptoms that I have already described, but they are even still more evident than in the second stage. The contraction of the iris is sometimes carried so far as entirely to close the pupil, and the irregularity of its contour becomes more and more manifest. Attached to the pupillary circumference are numerous filaments or fringes, which also greatly tend to modify its form, and the flakes of coagulable lymph contained in the pupil are in some instances so numerous as entirely to conceal it. When this is the case, they may present all the characters of false membranes, and give rise to false cataract. The colour of the pupillary zone undergoes considerable modification, the iris assuming in this region a grayish or yellowish tint. During the inflammatory period of the malady the congested state of the vessels renders the iris darker than usual, but these vessels becoming to a certain extent obliterated, the paler fluids alone remain. The change which takes place in the colour of the iris is not, therefore, to be attributed to the decomposition of the blood, but to the solidification of the lymph which has been effused.

It is more especially in this period of the disease that we meet with specks, ecchymosis, abscesses, purulent or sanguineous collections. Effusion of blood may take place in the tissue of the iris to such an extent as to reach from the larger or outer circumference of that organ to the pupillary margin. The iris is also often the seat of small abscesses, which may be easily recognised, especially when situated at the free edge of the pupil. They are often very numerous; indeed, I have seen as many as twenty-eight in the same eye. The synechia is more decided than in the second stage; and when the disease has arrived thus far, it has generally been long accompanied by inflammation of the cornea and conjunctiva. The symptoms which indicate that these affections have existed for a considerable length of time are, therefore, often observed. The alterations which occur in the aqueous humour might possibly lead a person unacquainted with the nature of the disease to believe in the existence of hypopion. Such an error, however, will never be committed by those who have practically studied the symptoms of iritis.

*The various forms of iritis.*—Iritis may, as I told you in a former lecture, occupy the anterior or posterior surface, or the parenchyma, of the iris; but the distinction between these forms of inflammation can only be made at the commencement of the disease, the extreme tenuity of the membrane not allowing inflammation to exist long in one region without extending to the remainder of the organ.

*Serous* iritis, in which the anterior or serous surface is the seat of inflammation, may be easily distinguished from uveal iritis. The surface of the cornea is bathed with fluid, and assumes a peculiar shining appearance which it does not present when the eye is in a healthy state. This peculiar appearance of the eye must not, however, be confounded with the epiphora or effusion of tears, which is met with in ulcerated keratitis. On raising the eyelids, which may be done without any difficulty, we merely find the eye presenting a moist, watery aspect. In ulcerated keratitis, on the contrary, the contraction of the eyelids is so great as often to render it nearly impossible to open the eyes, and as soon as tne cornea feels the contact of the air, the tears flow in abundance. In serous iritis the cornea also presents the greenish tint, but as the other symptoms of keratitis are absent, the nature of the disease can scarcely be mistaken. The surface of the inflamed membrane no longer appears smooth, as in the healthy state, and the transparency of the aqueous humour is modified. If you then examine the eye attentively, you will frequently perceive small specks or dots apparently situated on the internal surface of the cornea—a proof that the posterior lamella of that membrane is inflamed. This extension of the inflammation from the iris to the cornea is the necessary consequence of the anatomical disposition of the parts. The anterior chamber being lined by a serous membrane which exists both on the anterior surface of the iris and on the posterior surface of the cornea, it evidently follows that when one region of the membrane is inflamed, the inflammation must soon be propagated to the rest of the organ, as is nearly always the case with serous membranes. In pleurisy, for instance, if the inflammation begins by the costal pleura, it soon extends to the pulmonary pleura, and in peritonitis, if it begins by the parietal peritoneum, it soon extends to the visceral layer of that membrane. In this form of iritis the pain is but slight. If the inflammation persists, the aqueous humour contained in the anterior chamber increases, and the iris being pushed backwards, posterior synechia is produced. The anterior chamber then appears larger than usual.

In *uveal* iritis, the shining watery appearance of the cornea is not so well marked as in the preceding form of inflammation. The pain is more severe, of an inflammatory nature, and radiates to the different parts of the head and face. The greater violence of the pain may be easily accounted for when we consider that the uvea is prolonged on the retina, and is in immediate communication with the nervous system of the head. The pupil becomes troubled sooner in serous iritis: indeed, this form of inflammation is nearly always accompanied by more or less effusion of lymph in the pupil.

I shall not attempt to assign peculiar symptoms to *parenchymatous* iritis. It is extremely difficult, if not impossible, to separate it from the other two forms of inflammation. We may admit isolated inflammation of the surfaces of the iris, for we know that mucous or serous surfaces may be inflamed without the subjacent tissues participating in the inflammation, but in a membrane of such extreme tenuity as the one in question, it is scarcely possible for the parenchyma alone to be affected.

Did I not intend very shortly to treat at length the subject of specific ophthalmia, it would now be time to examine the various forms of inflammation which are described under the head of scrofulous, rheumatic, arthritic, and syphilitic sore iritis. Suffice it for the present to say that syphilitic iritis is, in my opinion, the only specific form of the disease which can be said to exist, and that all the other inflammatory affections which have been recognised by ophthalmologists ought to be erased from our nosological categories, as they only exist in the imagination of those who have described them. I shall, however, defer, for the present, the examination of the characters which warrant our recognising syphilitic iritis, merely observing that, although I admit the existence of this specific form of inflammation, I by no means recognise as symptoms of such an affection those characters which are generally considered to indicate its presence.

*Causes of acute iritis.*—The anatomical nature of the iris is such as alone satisfactorily to account for its being so frequently the seat of inflammation: indeed, it is probable that it would be much oftener inflamed than it actually is, were it not guaranteed, by the position it occupies, from numerous causes of inflammation to which the superficial tunics of the eye are exposed. The internal causes of iritis are not well known. Great influence has been ascribed to sudden change from heat to cold—to meteorological phenomena —in fine, to all those agents which are supposed to act injuriously on the economy. But these are general causes of disease, and not more likely to produce iritis than any other malady. We continually see persons pass from a warm country to a cold one, from a warm to a cold room, without being on that account attacked by iritis; but of ten persons who expose themselves to cold when in a state of perspiration, some will be attacked by one disease, and some by another; one, for instance, by rheumatism, another by pneumonia, another by iritis; whilst some will suffer no injury whatever. The appearance of one disease sooner than another, when we are exposed to the agency of these general causes, seems to depend on individual predisposition the nature of which we are not able to determine.

With the local causes of iritis we are much better acquainted. In operating for cataract by couching, the iris is often wounded, and iritis produced. This is also frequently the case when extraction is resorted to after the operation for artificial pupil, and in contused or punctured wounds of the eye. Some surgeons consider the iris to be endowed with extreme sensibility, and are alarmed at the very idea of touching it in the operations which are practised on the eye. Thus Beclard says that couching scarcely ever succeeds when the iris is touched with the needle, the mere contact giving rise to acute inflammation; and that were it possible to perform the operation without touching the iris, it would nearly always succeed. But when we operate for cataract by extraction, the crystalline lens, in passing through the wound of the cornea, must necessarily drag or tear the iris; and yet it is not so much iritis that is feared as the inflammation of the cornea which ensues, and the opacities by which that inflammation may terminate. Nor is the operation as unsuccessful as we should be inclined to consider, were we to adopt these opinions. Some practitioners say that they succeed in half or in three-fourths of the cases in which they operate. The efficacy of the method of operating which they adopt may have been exaggerated, it is true; as is nearly always the case when an exclusive opinion is professed: we are, nevertheless, authorized to conclude, from these statements, that what has been said of the exquisite sensibility of the iris cannot be perfectly correct. I have myself very frequently seen the iris cut or torn without its being consecutively inflamed. When, therefore, the iris becomes inflamed after local injury, the inflammation cannot be considered to depend entirely on the lesion it has received.

---

*Formation of an Artificial Anus.* By M. Amussat.—We recently reported a case in which M. Amussat had succeeded in establishing an artificial anus in the lumbar region of a lady affected with obstinate constipation. The same enterprising surgeon has more recently attempted a similar operation with nearly equal success.

M. T., 62 years of age, was affected with piles and constipation for several years. On examination of the rectum, it was discovered that a carcinomatous ulceration occupied the gut, about two and a half inches above the sphincter, nearly obliterating the cavity, and extending upwards for an inch and three-quarters. This state of the intestine was recognised by several medical men in consultation, and the means of alleviation were discussed. Dilatation and the ligature were rejected. Excision of the carcinomatous ring was also rejected, as any hæmorrhage might prove fatal to the patient, who was already reduced to the lowest state. After much deliberation, it was proposed to break up (broyer) the tumour, and this operation was performed by M. Amussat, on the 30th of May last, with a long pair of forceps, by which the most prominent granulations were crushed and removed. But little blood was lost, and the patient experienced hardly any pain. A current of cold water was now thrown into the rectum, to prevent, if possible, the development of inflammation; and, after the lapse of eight days, it was decided to cauterise the parts. Accordingly, M. Amussat applied the caustic potass at seven different times, by means of the speculum, an interval of three or four days being allowed to intervene between each application. No signs of inflammation about the bladder or peritoneum were thus produced, and the cancerous tumour was reduced to nearly one-half of its original volume. The patient's state, however, became gradually worse. An evacuation of the bowels took place only once every ten or twelve days, and was attended, each time, with a prostration of strength, often terminating in fainting fits. The skin covering the sacrum was on the point of ulcerating.

Under these discouraging circumstances, another consultation was held on the 13th of July, when it was unanimously determined to give the patient the chance of an operation for artificial anus. M. Amussat made an incision of four inches and a half in length, along the middle of the space comprised between the last false rib and the crista ilei, beginning at a distance of about four inches from the spinous processes of the vertebræ. The rest of the operation was completed in the manner which we have already described. At the anterior angle of the wound a membranous projection, formed by the peritoneum, presented itself, and beneath this seemed to lie the small intestines.

The sigmoid flexure of the colon was firmly contracted, and nearly covered by the quadratus lumborum muscle, the fibres of which were divided transversely. The intestine was now seized, with the necessary precautions, and the posterior half of the circumference divided. Some gas and scybalæ escaped. The colon was then drawn to the anterior edge of the wound, and fixed there by four points of suture. Three other stitches were applied to the edges of the wound, care being taken to leave the portion which corresponded with the gut perfectly free. The operation was not productive of any general accidents, but the opening did not immediately give passage to the fœcal matter, until the 18th of July, when an abundant evacuation took place through it. The opening was now gradually dilated by means of prepared sponge and bougies.

Since then the passage of fœces through the artificial anus has been considerably facilitated. The patient's general health has also so much improved, that he was able to return to the country; the hectic fever had disappeared, and a regular evacuation took place every day. On the 18th of August he was examined by M. Amussat and several other surgeons, who found that the disease of the rectum had not made any progress since the operation; and on the 22d of September, M. Amussat had a letter from the patient, announcing that he was able to walk about every day for an hour and a half, without any inconvenience.—*French Medical Gazette and Lancet.*

---

*Ægilops and triticum.*—Some botanists have supposed, from the extreme resemblance of the fruit of the ægilops to the grains of cultivated wheat, that the latter is only an ægilops modified by cultivation. M. Esprit Fabre, whose excellent observations on the fructification of the marsilea are known to every botanist, having found some plants of the ægilops triticoïdes in the neighbourhood of Agde, last year, has sown the fruit in his garden, and has obtained a plant in which the characters of the ægilops have almost entirely disappeared and made room for those of the triticum.—*Gazette Médicale*, Aug. 17, 1839.

